# Quality of life measures in Italian children with atopic dermatitis and their families

**DOI:** 10.1186/1824-7288-37-59

**Published:** 2011-12-22

**Authors:** Fiorella Monti, Francesca Agostini, Francesca Gobbi, Erica Neri, Sandra Schianchi, Fabio Arcangeli

**Affiliations:** 1 Department of Psychology, University of Bologna, Viale Berti Pichat 5, Bologna, Italy; 2 Dermatology Unit, M. Bufalini Hospital, Cesena, Italy

## Abstract

**Background:**

The impact of atopic dermatitis (AD) on children's quality of life (QoL) in US and European countries is relatively well known, though rarely evaluated in the Italian population. Moreover, the association between child age and QoL has not been enough investigated, even though few studies detected a worse QoL in youngest AD children. The aim of the study was to evaluate the QoL in an Italian sample of atopic children and their families, also exploring a possible association with child age.

**Methods:**

60 AD children aged between 1-12 years and their mothers completed specific QoL questionnaires (IDQoL/CDLQI, DFI) and a clinician completed a measure of AD severity (SCORAD).

**Results:**

AD severity (Objective SCORAD) significantly correlated with QoL measures. Severe AD children showed higher IDQoL/CDLQI and DFI scores compared to mild and moderate AD groups (*P *= 0.006 and *P *< 0.0005, respectively), but only DFI scores differed in these last two conditions (*P *= 0.014). DFI scores negatively correlated with children's age (*P *= 0.046), but did not differ when considering child age ranges. Multiple linear regression analyses revealed a significant association between Objective SCORAD and QoL measures.

**Conclusions:**

A strong association between severe AD and poor QoL, both in children and mothers, was found in the Italian sample, in line with the international literature. Family's QoL scores were sensitively related to AD severity, more than the child's QoL, emphasising that the disease has a deep impact on the family. A significant association between age and QoL was only partially found and needs further investigation.

## Background

Atopic dermatitis (AD), also known as atopic eczema, is one of the most common chronic inflammatory skin disease which occurs during childhood, affecting 10-20% of children in Europe [1], and 17% of children in the United States [2]. Time trends in atopy have shown a substantial increase since the early 1960s and there is evidence of consistent associations of these disorders with a Western lifestyle [3].

Usually childhood AD onsets in the first five years of life, with about 60% of cases appearing within the first year, frequently between 0 and 6 months [4].

Adverse consequences of this disorder on children, like distress, irritability, behavioural problems and sleep dysfunctions, have been reported in many previously published articles [5-8]. Besides, negative influences have been observed in families, such as frustration, feelings of inadequacy about their role as parents, concerns, fears about the disease, self-blame and disappointments [4,7,9-14].

Recently, the detection of these adverse consequences have led to estimate the AD impact on the young patient's Quality of Life (QoL) [12,15-19], putting in evidence how a bad skin condition is frequently associated to a poorer QoL.

QoL is a wide concept, influenced by physical health, psychological state, level of independence and social relations. The World Health Organization (WHO) has tried to give quality of life (QOL) an exact definition: "The individual's perception of his position in life in the context of culture and value systems in which he lives and in relation to his goals, expectations, standards and concerns" [20].

In medicine, attempts to construct methods for measuring QOL have primarily focused on individuals with chronic diseases with elevated costs of care and treatment, in order to better evaluate the impact of the disease on patients. A recent study [6] found that atopic children had a worse QoL than children affected by other chronic diseases (like, for example, naevi, acne, alopecia, diabetes, psoriasis, asthma, cystic fibrosis, generalised eczema), except cerebral palsy. Moreover, some studies evaluated the association between child's age and QoL, where a major impact was observed in children of youngest age [21,22] and their families [21].

While several studies have explored prevalence of AD and related QoL in childhood, in USA and European countries, to our knowledge few published studies have analysed these aspects in Italian children [8,23,24].

The present study is a cross-sectional analysis, the aim of which was to investigate the impact of AD on the QoL of an Italian sample of atopic children and their families. The expected result is to find an association between a bad skin condition and a poorer QoL in children and their parents. Secondly, the study aimed at exploring a possible association between child's age and child's and family's QoL.

## Patients and Methods

### Study Subjects

During the period March 2006-March 2007, all consecutive AD patients (aged 1-12 years), attending the Dermatology Unit of M. Bufalini Hospital, Cesena (Italy), and their mothers were considered eligible for the study and therefore asked to participate.

AD patients were enrolled in the study during a routine check-up in the Day-Hospital by a dermatologist, who registered the clinical profile and calculated the objective SCORAD, following the diagnostic criteria proposed by Hanifin and Rajka [25]. A psychologist then presented the research project to the mothers and their children; all subjects who were asked to participate consented to take part in the study and received the informed consent form and a range of questionnaires to complete: Infants Dermatitis Quality of Life Index (IDQoL), Children's Dermatology Life Quality Index (CDLQI), Dermatitis Family Impact (DFI). All questionnaires were completed entirely. For children aged 5-12 years, all the mothers completed the DFI, while their children completed the CDLQI; younger children's mothers helped them in understanding and filling in the questionnaires. For the children aged 1-4 years old, all mothers completed the DFI and the IDQoL.

Ethical approval (n° PR 887026) for the study was obtained from the Hospital Ethics Committee.

### Instruments

The instruments used in the study were:

The SCORAD [26], an index aimed at assessing AD disease severity, where the higher the value the worse the skin condition. Given that in literature [27] it has been demonstrated that subjective symptoms do not always correlate with disease severity and objective assessment, and in relation to the recommendations by Oranje et al. [27], we used the objective SCORAD, instead of the SCORAD index, including extent and intensity of the lesions, divided into three levels: < 15 mild, 15-40 moderate, > 40 severe [27].

A questionnaire including items on the main socio-demographic characteristics.

The Infants Dermatitis Quality of Life Index (IDQoL [28]; Italian validated version [29]), a disease-specific measure for AD children between 0-4 years old, consisting of 10 items concerning physical and social functioning and completed by the caregiver. A total score is calculated (range 0-30), where higher scores represent a poorer quality of life.

The Children's Dermatology Life Quality Index (CDLQI), a skin-related measure for children between 5-16 years old [30], consisting of 10 items on physical and social functioning. The total score is between 0 and 30, where higher scores represent a poorer quality of life.

The Dermatitis Family Impact questionnaire (DFI [13]; Italian validated version [29]), a 10-item scale measuring the impact of AD on families with a child affected by the disease, validated on a parents' sample of AD children aged 6 months-10 years old; the total score is calculated and ranges from 0 to 30, where, again, the higher the scores the poorer the quality of life.

### Statistical analysis

Disease severity groups (mild, moderate, severe) were defined in function of the objective SCORAD ranges; patients were also classified in respect to specific age groups (1-4, 5-7 and 8-12 years), which were chosen considering main developmental stages [31,32].

Qualitative variables were compared using Chi Square test. Quantitative variables were analyzed first of all using correlations (Pearson's coefficient) and then using ANOVA; specifically, HRQoL scores of participants in different groups (according to AD severity levels and age groups) were compared using ANOVA and performing post hoc analyses with Tukeys Honestly Significant Difference (HSD) test. A multiple regression was performed in order to verify how much each variable contributed to an explanation of the HRQoL scores. All the following variables were included as predictors: child's gender, child's age, Objective SCORAD, presence of other allergies, caregiver's age, marital status and socio-economic level. Considering the low number of subjects, the Enter method was chosen as the safest one for the regression.

Statistical analyses were performed using the SPSS Package for Windows software, version 17.0.

## Results

### Clinical characteristics of the study subjects

A total of 60 AD children (range 1-12 years; mean ± SD: 4.5 ± 3.3), including 30 females and 30 males, and their 60 mothers were recruited (range 25-50 years old; mean ± SD: 36.5 ± 5.8). Considering child age range, 37 children (61.7%) were between 1 and 4 years old, 12 (20%) between 5-7 years old and 11 (18.3%) between 8-12. In 16.7% of cases, the children also suffered from another form of allergy, specifically cases of food allergy.

The majority of mothers were married (89.6%); most of them had a permanent job (78.3%): 48.3% of them were employees, 10% were self-employed and 20% worked in other fields. According to Hollingshead criteria for socioeconomic status [33], women were classified as belonging to one of three levels: low (27.1%), medium (60.4%) and high (12.5%).

The mean Objective SCORAD score of the total sample was 29.46 (± 16.31; range 3.5-74); no significant differences emerged in the mean objective SCORAD score among the 3 children's age groups (*P *= 0.93): children between 1-4 years old had a score equal to 28.81 (± 14.85; range 3.5-59.5), 5-7 year old children scored 30.49 (± 19.52; range 10-74), while 8-12 year old children scored 30.51 (± 18.78; range 8.8-62), (Table 1).

**Table 1:** Objective SCORAD scores of 60 Children

	AD Children			
	**1-4 years (*n *= 37)**	**5-7 years (*n *= 12)**	**8-12 years (*n *= 11)**	**p-value**

**Objective SCORAD (mean ± SD)**	28.81 ± 14.85	30.49 ± 19.52	30.51 ± 18.78	0.93
**Objective SCORAD severity**	-	-	-	
**Mild**	10 (27.0)	3 (25.0)	3 (27.3)	0.97
**Moderate**	18 (48.6)	7 (58.3)	5 (45.5)	
**Severe**	9 (24.3)	2 (16.7)	3 (27.3)	

Figures in parentheses are percentages.

According to the categories of the objective SCORAD, 26.7% (16) of children showed a mild AD, 50% (30) a moderate AD and 23.3% (14) a severe AD. The distribution of children across AD severity levels and age ranges did not show any significant difference (χ^2 ^= 0.559, df = 2 *P *= 0.97), (Table 1).

Also, the distribution of women in function of AD severity and levels of socio-demographic variables did not show significant differences (marital status: *P *= 0.25; job: *P *= 0.34; socioeconomic status: *P *= 0.64).

### Correlations among SCORAD and QoL measures

Correlations between Objective SCORAD and IDQoL/CDLQI and DFI scores were all statistically significant, even though moderate (*r *= 0.401, *P *= 0.001 and *r *= 0.414, *P *= 0.001, respectively). The correlation between DFI and IDQoL/CDLQI was high and significant (*r *= 0.755, *P *< 0.0001). Child's age negatively correlated with DFI scores (*r *= -0.258, *P *= 0.046), but not with Objective SCORAD (*r *= 0.098, *P *= 0.46) nor with IDQoL/CDLQI (*r *= -0.119, *P *= 0.367).

### Quality of life in relation to disease severity and child's age: comparisons among patient groups

IDQoL/CDLQI mean score in the total sample was 7.0 ± 5.21 (range 0-19). According to AD severity (Objective SCORAD categories), IDQoL/CDLQI mean scores appeared to be statistically different (*P *= 0.006): post hoc analyses showed that the group with severe AD got a higher score compared to the group with mild (*P *< 0.0005) and moderate AD (p = 0.038), but these last two were not statistically different (*P *= 0.063; Table 2). When considering child's age range, differences in IDQoL/CDLQI mean scores did not emerge among groups (*P *= 0.28; Table 2), not even when looking at the interaction between AD severity and age range (*P *= 0.87).

**Table 2:** QoL measures in relation to AD severity and child's age

		Objective SCORAD		
	Mild (*n *= 16)	Moderate (*n *= 30)	Severe (*n *= 14)	p-value

**IDQoL/CDLQI, mean ± SD**	3.63 ± 4.03	7.0 ± 4.70	10.86 ± 5.02	0.006
**DFI, mean ± SD**	3.38 ± 5.02	8.1 ± 5.30	12.79 ± 5.30	< 0.0005

		**Age groups**		

	1-4 years (*n *= 37)	5-7 years (*n *= 12)	8-12 years (*n *= 11)	p-value

**IDQoL/CDLQI, mean ± SD**	7.57 ± 5.02	6.83 ± 4.17	5.27 ± 6.77	0.28
**DFI, mean ± SD**	9.32 ± 6.37	6.08 ± 5.52	5.36 ± 5.41	0.074

Data are means ± SD

DFI mean score in the total sample was 7.95 ± 6.21 (range 0-23). DFI mean scores as well differed in relation to AD severity levels (*P *< 0.0005): the group with severe AD showed a higher score compared to mild (*P *< 0.0005) and moderate AD groups (*P *= 0.024) and the latter was significantly higher than the mild group (*P *= 0.014; Table 2). Further significant differences did not emerge when considering the child's age range variable (*P *= 0.074; Table 2), nor when looking at "AD severity x child's age" (*P *= 0.99).

### Multiple linear regression of variables associated with QoL measures

Using the Enter method, a significant model emerged (*P *< 0.037), in which 22% (Adjusted R^2 ^= 0.217) of the variance in IDQoL/CDLQI scores was explained by Objective SCORAD (Beta coefficient = 0.479, *P *= 0.003), while all the other considered variables (sex, child's age, food allergies, mother's age, marital status, socio-economic level) did not significantly contribute to the variance (Table 3).

**Table 3:** Contribution of sociodemographic and clinical variables to QoL measures

	Predictor Variables	β values	*t*	p-value
**IDQoL/CDLQI scores**	Sex	-0.158	-1.067	0.294
	Age	-0.122	-0.624	0.537
	Objective SCORAD	0.479	3.190	0.003
	Food allergies	-0.197	-1.242	0.223
	Mother's age	0.198	1.012	0.319
	Marital status	-0.195	-1.249	0.221
	Socio-economic level	0.144	0.963	0.348

**DFI scores**	Sex	-0.197	-1.375	0.179
	Age	-0.174	-0.943	0.363
	Objective SCORAD	0.558	3.958	0.000
	Food allergies	-0.205	-1.375	0.179
	Mother's age	0.013	0.071	0.944
	Marital status	-0.058	-0.398	0.694
	Socio-economic level	-0.001	-0.010	0.992

Considering DFI scores as criterion variable, a significant model emerged too (*P *< 0.008); again, Objective SCORAD (Beta coefficient = 0.558, *P *< 0.0005) appeared to be the only significant predictor, explaining 31% of the variance (Adjusted R^2 ^= 0.308) (Table 3).

## Discussion

To our knowledge, the current study is one of the few published investigating QoL in an Italian sample of children with atopic dermatitis.

Globally, the results showed a strong association between QoL and disease severity, measured by Objective SCORAD, as already shown by international literature [16,17,19]. In fact, the study found that a bad skin condition was significantly associated with a poorer child QoL and with a significant worsening of family's QoL, evidencing how AD tends to affect the whole family system and not only the individual patient. Compared to a similar Italian study by Ricci et al. [8], including 45 AD children aged between 3 months-7 years old, our children and families' QoL scores were noticeably lower, but we must consider that a higher percentage of severe AD was present in that sample (44.4% severe AD children vs 23.3% in our study).

Looking into the details of our results and considering both individual patients' and families' QoL scores, we found some similarities and differences between the two.

First of all, both QoL scores showed a moderate correlation with Objective SCORAD, reporting very similar coefficients; the scores also showed to be significantly higher in severe AD children, compared to moderate and mild AD conditions. That is, poorer QoL was strictly associated to a more serious AD condition.

At the same time, differences between patients' and families' perspectives emerged in relation to the following aspects: while IDQOL/CDLQI scores were almost equal in moderate and mild AD children, DFI scores showed significant differences across all the 3 levels of AD severity. This means that, in our sample, families' QoL appeared to be noticeably affected by the intensity and severity of the child disease, more than the individual QoL. This aspect emphasises the main role played by the family in the management of the disease and should be more frequently kept in mind by clinicians.

Another difference that emerged between IDQOL/CDLQI and DFI measures was that only the latter appeared to be associated with child's age. In fact, DFI scores showed a negative correlation, even if weak, with the child's age: the parents of younger AD children seemed to experience a poorer QoL, as already suggested in the study by Ganemo et al. [21]. Anyway, given the small size of our sample, the non significant *P *value detected when considering DFI scores in relation to different age groups (*P *= 0.074) and the non-relevance of the child's age as predictor variable in regression analysis, more investigations are needed.

The study has nevertheless some limitations. First of all, difficulties in recruiting the subjects have resulted in a limited sample size and a reduced number of considered variables and, because of this, further analyses on wider Italian samples are needed in future to obtain more statistically and clinically relevant data. Secondly, we had no control group and this did not allow the comparison between our results and the QoL of healthy children or children affected by other dermatological diseases. In future studies, therefore, it would be relevant to investigate whether in the Italian population the trends shown in literature, where AD subjects exhibit poorer QoL compared to control groups or other clinical groups, are confirmed [6,19].

## Conclusions

However, globally our data support the main clinical implications derived from recent international studies on QoL in atopic children; as underlined by Brenninkmeijer et al. [34], "in cases of severe AD, dermatologists should not only be attentive to the physical aspects but also to the psychological and social aspects of AD. In conclusion, we believe that in clinical care a systematic evaluation of physical and psychosocial consequences in patients with AD is warranted". Understanding HRQoL in the patient and his/her family might help professional operators to improve their relationship with them and to facilitate the management of treatment regimes. While early investigations tended to focus on socio-demographic and clinical variables, recent studies have shown that there are multiple factors which determine adherence, such as personal perception of health status, coping style, worries, burden, social reasons, motivation and side effects of treatments [6,35]. Warschburger et al. [36] have underlined how parental disease management is predicted by familial situation, personal well-being and severity of child disease.

For these reasons, it would be useful in future studies to focus on psychosocial and psychological variables that could affect families' QoL, such as analysing whether there is a substantial difference in mother and father's perceptions, as investigated by Holm et al. [37] and Chernishov [38], or if comorbidity with other parents' diseases can have some influence on their ability to provide the child with adequate care. At the same time, studies examining the effects of education programs on parental support should be promoted [39].

## Competing interests

The authors declare that they have no competing interests.

## Authors' contributions

FM and FG conceived the study and coordinated it. FA and EN performed the statistical analysis and drafted the manuscript. SS and FAR participated in the design of the study.
